# Amount of Gold Foil Used in the United States for Filling Teeth

**Published:** 1851-01

**Authors:** 


*Amount of Gold Foil used in the United States for Filling Teeth.*—We have been at some pains to ascertain the gross amount of gold foil used annually in the United States, which, as nearly as can be learned, is about 6,600 ounces, which, at $ 30 per ounce, about the average selling price, would amount to $ 198,000.

